# Linking Ecology and Epidemiology to Understand Predictors of Multi-Host Responses to an Emerging Pathogen, the Amphibian Chytrid Fungus

**DOI:** 10.1371/journal.pone.0167882

**Published:** 2017-01-17

**Authors:** Stephanie S. Gervasi, Patrick R. Stephens, Jessica Hua, Catherine L. Searle, Gisselle Yang Xie, Jenny Urbina, Deanna H. Olson, Betsy A. Bancroft, Virginia Weis, John I. Hammond, Rick A. Relyea, Andrew R. Blaustein

**Affiliations:** 1 Monell Chemical Senses Center, Philadelphia, Pennsylvania, United States of America; 2 Odum School of Ecology, University of Georgia, Athens, Georgia, United States of America; 3 Biological Sciences Department, Binghamton University, Binghamton, New York, United States of America; 4 Department of Biological Sciences, Purdue University, West Lafayette, Indiana, United States of America; 5 Department of Integrative Biology, Oregon State University, Corvallis, Oregon, United States of America; 6 Environmental Sciences Graduate Program, Oregon State University, Corvallis, Oregon, United States of America; 7 United States Forest Service, Pacific Northwest Research Station, Corvallis, Oregon, United States of America; 8 Biology Department, Gonzaga University, Spokane, Washington, United States of America; 9 Department of Biology, University of New Mexico, Albuquerque, New Mexico, United States of America; 10 Department of Biological Sciences, Rensselaer Polytechnic Institute, Troy, New York, United States of America; Universitat Trier, GERMANY

## Abstract

Variation in host responses to pathogens can have cascading effects on populations and communities when some individuals or groups of individuals display disproportionate vulnerability to infection or differ in their competence to transmit infection. The fungal pathogen, *Batrachochytrium dendrobatidis* (Bd) has been detected in almost 700 different amphibian species and is implicated in numerous global amphibian population declines. Identifying key hosts in the amphibian-Bd system–those who are at greatest risk or who pose the greatest risk for others–is challenging due in part to many extrinsic environmental factors driving spatiotemporal Bd distribution and context-dependent host responses to Bd in the wild. One way to improve predictive risk models and generate testable mechanistic hypotheses about vulnerability is to complement what we know about the spatial epidemiology of Bd with data collected through comparative experimental studies. We used standardized pathogen challenges to quantify amphibian survival and infection trajectories across 20 post-metamorphic North American species raised from eggs. We then incorporated trait-based models to investigate the predictive power of phylogenetic history, habitat use, and ecological and life history traits in explaining responses to Bd. True frogs (*Ranidae*) displayed the lowest infection intensities, whereas toads (*Bufonidae*) generally displayed the greatest levels of mortality after Bd exposure. Affiliation with ephemeral aquatic habitat and breadth of habitat use were strong predictors of vulnerability to and intensity of infection and several other traits including body size, lifespan, age at sexual maturity, and geographic range also appeared in top models explaining host responses to Bd. Several of the species examined are highly understudied with respect to Bd such that this study represents the first experimental susceptibility data. Combining insights gained from experimental studies with observations of landscape-level disease prevalence may help explain current and predict future pathogen dynamics in the Bd system.

## Introduction

Understanding the responses of different hosts to generalist pathogens is essential for making accurate predictions about species persistence and pathogen spread in ecological communities [1–3]. Hosts vary both in the way they encounter and are exposed to pathogens, and in the way they respond to infection [4–5]. Variation in exposure is mediated by biotic and abiotic factors that drive the spatiotemporal distribution and abundance of hosts and pathogens in the environment. Further, the ecology, life history and behavioral traits of hosts can influence the probability and frequency of contact with pathogens as well as the outcome of and potential to transmit infection once contact occurs [5–8]. Post-exposure heterogeneity in host susceptibility (propensity to become infected), vulnerability (risk of mortality), infectiousness (intensity of infection or infection load), and duration of infection are mediated through a combination of genetic, molecular, physiological and behavioral processes that arise at the level of the individual [9]. Differences in host responses can subsequently drive disease dynamics. For example, some individuals [10–12], species [13–15] or taxa [6] may have disproportionate effects on pathogen spread and persistence. Identifying hosts that dilute or amplify pathogen transmission [15] may allow for more efficient pathogen management and species conservation, which is of central importance as the number and severity of emerging infectious diseases increase globally [16].

The amphibian-chytrid fungus system is ideal for investigating host-pathogen variation. The emerging infectious fungal pathogen, *Batrachochytrium dendrobatidis* (Bd), is thought to be responsible for the most spectacular loss of vertebrate biodiversity due to disease in recorded history [17]. Bd is found on every continent where amphibians exist, infects almost 700 different amphibian species globally [18–20] and is associated with worldwide amphibian population declines, range reductions and species extinctions (e.g., [21–22]). One recent projection based on IPCC Climate Futures suggests that environmental suitability for Bd will expand in the temperature zones of the Northern Hemisphere; specifically, under predicted climate change, Bd ranges are expected to shift into higher latitudes and altitudes [23]. Not all species or populations show declines when Bd is present [24–26] and susceptibility and vulnerability of many species is still relatively unknown. Several studies highlight a disconnect between predicted environmental suitability for Bd (*i*.*e*., risk of host exposure) and the detection of population level declines due to the fungus [27–28] suggesting that risk of negative population-level outcomes is also largely dependent on intrinsically-mediated host traits such the ability to tolerate or resist Bd [29–32].

Our goal in this study was to obtain a broad view of host variation in the amphibian-Bd system and generate experimentally-derived estimates of vulnerability for several understudied species. Therefore, we investigated host responses to standardized Bd-exposure across 20 North American amphibian species, encompassing three major amphibian families (Ranidae, Bufonidae and Hylidae). All animals were reared from the egg stage to metamorphosis to control for confounding effects of previous pathogen exposure and larval environment. We then used our experimental estimates of host vulnerability and infection intensity as response variables in phylogenetically-informed predictive models to identify key ecological and life history variables associated with host responses to the fungus. We compare our top predictors of risk with those identified in prior studies and summarize how our results might inform existing strategies for disease management and amphibian conservation.

## Results

### Survival

**Fig 1:**
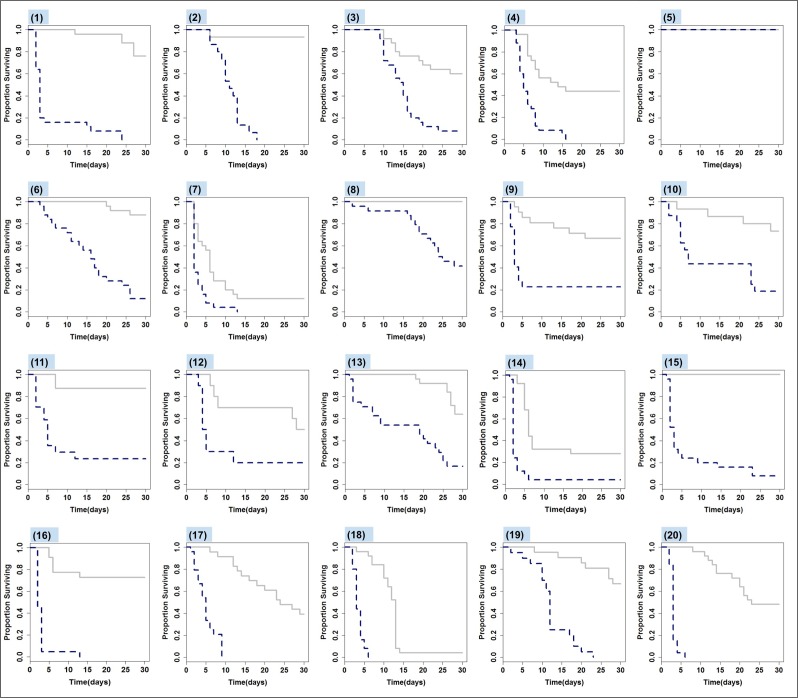
Kaplan-Meier survival curves for 20 amphibian species exposed to *Batrachochytrium dendrobatidis* (blue dashed lines) or in control treatments (gray solid lines) over 30 day experimental trials. Species are grouped top to bottom by family (*Hylidae*, tree frogs = *Pseudacris* and *Hyla*; *Ranidae*, true frogs = *Rana* and *Lithobates*; *Bufonidae*, toads = *Anaxyrus*). Numbers listed at the top of each survival curve correspond to the following species: 1 –Pseudacris regilla*, 2 –Pseudacris ornata*, 3 –Pseudacris crucifer*, 4 –Pseudacris feriarum*, 5 –*Hyla squirella*, 6 –Pseudacris triseriata*, 7 –Hyla versicolor*, 8 –Hyla wrightorum*, 9 –Rana aurora*, 10 –Rana cascadae*, 11- Lithobates catesbeianus*, 12 –Lithobates clamitans*, 13 –Rana luteiventris*, 14 –Lithobates pipiens*, 15 –Lithobates sphenocephalus*, 16 –Lithobates sylvaticus*, 17 –Anaxyrus fowleri*, 18 –Anaxyrus americanus*, 19 –Anaxyrus boreas*, 20 –*Anaxyrus terrestris*. Species names followed by an asterisk indicate a significant within-species treatment effect (Control versus Bd survival in the Cox Proportional Hazards model without covariates, at p ≤ 0.05). Survival curve for species 11 reprinted with permission from Gervasi et al. 2013, Ecohealth [38] Rights Link license number 3915511271582. Survival curves for species 4, 6–7, 14, 16 and 20 reprinted with permission from Searle et al. 2011, Conservation Biology [32], Rights Link license number 3915510850508.

**Fig 2:**
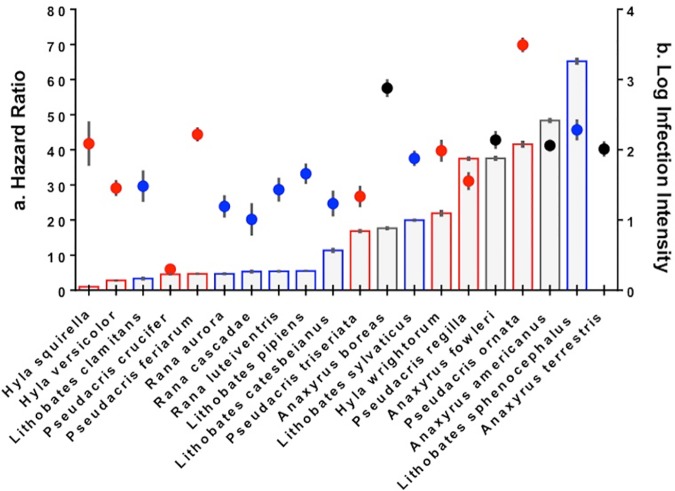
**(A) Hazard ratios displayed with bars and (B) average infection loads displayed with circle symbols for 20 amphibian species experimentally exposed to *Batrachochytrium dendrobatidis*.** Hazard ratios represent the difference in risk of mortality between the Bd-exposed and control treatments. Hazard ratios > 1 indicate greater risk of mortality associated with the Bd treatment. Species are listed from lowest to highest hazard ratio from left to right and *H*. *squirella* was the only species that did not show a significant within-species treatment effect (HR = 1). Hazard ratios were obtained from Cox Proportional Hazards models that were fit without covariates, which explains why exact values differ from those previously reported [32, 38]. Infection load values represent average Bd zoospore genome equivalents detected in the skin of animals across the entire duration of the experiment (calculated as log_10_ (1 + average load) for each species. Error bars (on bars and points) = +/- 1 SE of the mean. Black dots and bar outlines indicate species in the family *Bufonidae*, red dots and bar outlines correspond to species within the family *Hylidae* and blue dots and bar outlines represent species in the family *Ranidae*.

Survival ranged from 0 to 100% among amphibian species exposed to Bd, hazard ratios ranged from 1 to 65, and log response ratios ranged from -0.244 to -2.09 (more negative LRRs indicate a larger negative effect of Bd-treatment on survival; Fig 1, Fig 2A, S1 Table). Significant treatment differences (Bd versus Control hazard ratios > 1 and treatment difference significant at p ≤ 0.05) were observed in every species except *Hyla squirella*. In 13 of 20 species examined, mortality of Bd-exposed animals was greater than 50% within the first week of the 30d experiment (S1 Table). Overall, treatment and species identity were significant predictors of number of days survived in the experimental trial (*X*^*2*^_*1*_ = 530.8, P < 0.0001 for treatment; *X*^*2*^_*18*_ = 357.2, P < 0.0001 for species identity) and a significant interaction between treatment and species identity (*X*^*2*^_*1*_ = 109.0, P < 0.0001) indicated that effects of the pathogen treatment on survival varied among species (Fig 1, Fig 2A). Mass also predicted survival (*X*^*2*^_*1*_ = 47.07, P < 0.0001), but the effect of mass depended on treatment affiliation (interaction *X*^*2*^_*1*_ = 18.86, P < 0.0001, with a positive effect of mass on survival time in the Bd but not the control treatment) and species identity (interaction *X*^*2*^_*1*_ = 57.86, P < 0.0001). The effect of mass on survival was positive and significant (all P < 0.05) in 8 of 20 species (5 Ranid species, 2 Hylids, and 1 Bufonid; S1 Table). We further explored the role of body mass as a predictor of host vulnerability in our species-level ecological models.

We removed *Hyla squirella* from Cox proportional hazards models, as this species experienced 100% survival in both the Bd-exposed and Control treatment (thus, a HR of 1; Fig 1). We also excluded *Anaxyrus terrestris* from survival analyses because of the high number of ties in the date of mortality, which prevented us from using the Cox regression model and obtaining a meaningful hazard ratio [33]. We observed variation in rates of mortality of control animals in our study, which was not entirely unexpected given that our study was designed to explore responses of newly metamorphosed amphibians. Metamorphosis is a highly vulnerable developmental stage when mortality can be high and susceptibility to infection can be elevated [34–37]. Mortality at the metamorphic stage is naturally variable among species and it is also possible that species differed in intrinsic sensitivities to our experimental protocol (e.g., rearing conditions in mesocosms and experimental conditions in the laboratory). We observed no pathological signs of infection in control animals who died during the experiment, but we did not perform molecular diagnostics on these animals for any pathogens other than Bd. All control animals sampled tested negative for Bd. Importantly, the metrics used to quantify survival in this study (hazard ratio and log response ratio) were calculated by taking into account the differences in survival between experimental controls and Bd-exposed individuals. In addition, we provide separate analyses for both the full set of 20 species, as well as a reduced set of species (e.g., excluding 3 species that experienced the highest levels (≥ 30%) mortality in controls).

### Infection Intensity

Infection intensities (average Bd infection loads) in the skin of amphibians exposed to standardized inoculations of the fungus spanned over 3 orders of magnitude (Fig 2B, S1 Table). Species identity and number of days survived were significant predictors of infection intensity (F_19,387_ = 30.38, P < 0.0001 for species identity; F_1,387_ = 98.43, P < 0.0001 for days survived), and overall, longer persistence over the 30-d trial was associated with lower infection intensities. There was no main effect of mass on infection intensity (F_1,387_ = 1.10, P = 0.2942), but the relationship between mass and infection intensity varied depending on species identity (interaction term F_1,19_ = 2.566, P = 0.0003). In 7 of 20 species (5 Ranid and 2 Hylid species) initial body mass was a significant predictor of average infection intensity over the experiment (all P < 0.05); in all but 1 of these species (*Hyla versicolor*) smaller body mass was associated with higher experimental infection intensities (S1 Table). In *Hyla versicolor*, smaller initial body mass was associated with lower average infection intensity.

The highest average infection load was observed in *Pseudacris ornata* (average untransformed value = 4.5 X 10^3^ genome equivalents; max untransformed value = 1.2 X 10^4^ genome equivalents) whereas the lowest average infection load was observed in *Pseudacris crucifer* (average untransformed value = 1.41 genome equivalents; max untransformed value = 5.35 genome equivalents, Fig 2A, S1 Table). Bd-positive individuals were observed in every species in this study. In fact, in 13 of 20 species, every Bd-exposed individual also tested positive for Bd (S1 Table). However, in 7 of 20 species a percentage of individuals (ranging from 4–19% of Bd-exposed animals) tested negative for Bd infection, even after repeated weekly experimental inoculations (S1 Table). In all of these cases, individuals who tested negative for Bd infection also survived to the end of the experiment (though not all individuals surviving to the end of the experiment were infection-negative). When we examined a subset of all animals who died within the first week of exposure, we already could detect a significant difference in infection intensity at the species level (F_17,245_ = 7.04, P < 0.0001), suggesting that, as with survival, a single inoculation with Bd was enough to drive significant species-level variation in infection intensity.

### Phylogenetic Comparative Analyses

**Fig 3:**
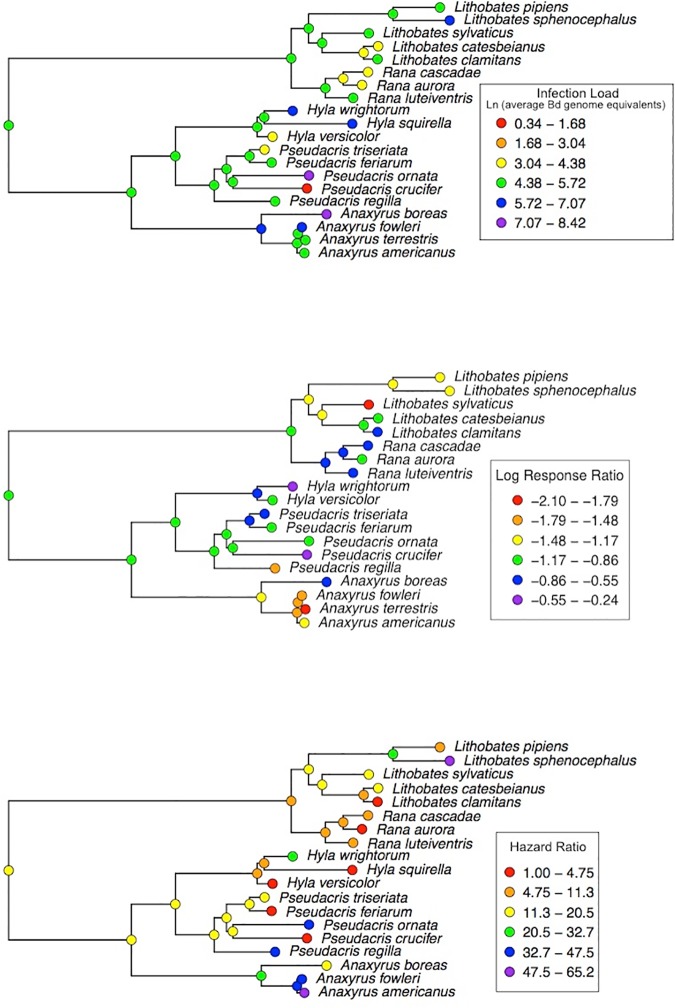
Phylogenetic reconstructions for response variables. (**A**) average infection load displayed as the natural logarithm of average *Batrachochytrium dendrobatidis* genome equivalents detected in amphibian skin after exposure to the pathogen, (**B**) log response ratio, displayed as the effect size for the difference in survival between the Bd and Control treatments, and (**C**) hazard ratio, describing the risk of mortality in the Bd treatment compared to the control treatment.

We observed a significant phylogenetic signal for average infection load (P = 0.033), a marginally significant phylogenetic signal for hazard ratio (P = 0.056) and no significant phylogenetic signal for log response ratio (P = 0.118, Fig 3A–3C; Table 1). Differences in predictor variables among species showed a significant tendency to scale with the phylogenetic distance among species (Table 1). For response and predictor variables, significant signal was more frequently observed under the gradual compared to the speciational model of character evolution (Table 1). We therefore conducted phylogenetic generalized least squares (PGLS) analyses assuming a gradual model of character evolution. Maximum likelihood estimates of lambda (the amount of phylogenetic adjustment to incorporate into a regression model) during PGLS analyses were always zero, indicating no adjustment for phylogeny (S2 Table). This was most likely due to a lack of phylogenetic signal in model residual errors, despite the weak signal that some traits showed [39] in most of our explanatory variables (Table 1).

**Table 1:** Phylogenetic signal as measured by Blomberg's K and associated significance values and transformations performed on response and explanatory variables to meet assumptions of linearity in predictive models. Two phylogenetic trees were used to quantify phylogenetic distance among species. K (Speciational) was based on previous studies of anuran phylogeny and assumed a speciational model of trait evolution. The other tree, K (Gradual) was estimated via maximum likelihood from 2500 bp of mitochondrial sequence data obtained from GenBank.

Factor	K(Gradual)	P value	K(Speciation)	P value	transformation
Infection intensity	0.299	**0.033**	0.393	0.718	Log
Hazard ratio	0.300	**0.056**	0.426	0.463	^0.5
Log response ratio	0.203	0.118	0.515	0.129	None
Average mass at metamorphosis	0.348	**0.006**	0.960	0.002	Reciprocal root
Habitat central tendency	0.519	**0.001**	0.924	0.001	None
Habitat breadth	0.362	**0.009**	0.761	0.007	None
Median adult size/snout-vent length	0.944	**0.001**	1.944	0.001	Log
Median larval period	0.606	**0.001**	1.069	0.001	Log
Median age at sexual maturity	0.203	0.141	0.862	0.008	Log
Median lifespan	0.202	0.177	0.779	0.012	Reciprocal root
Median eggs per year	0.324	**0.006**	0.727	0.012	Log
Geographic range size	0.153	0.332	0.427	0.482	^0.33333333

Caret symbol (^) indicates exponentiation.

There were 6 initial candidate PGLS models for average infection load (as determined by delta AICc scores less than 2 ([40], S3A Table). Three of these models were excluded from selection because they had additional terms that did not improve the AICc score of the more complex model, compared to the simpler candidate model [41]. S3 Table (A) shows all infection load candidate models and the top 3 models (highlighted in yellow). Two of the 3 selected top models included habitat use (habitat central tendency) as a significant predictor of infection intensity across species. A negative linear relationship between habitat use and infection intensity indicated that more ephemeral habitat usage (smaller score, S3 Table A) was associated with higher Bd infection loads. Conversely, use of permanent aquatic habitats was associated with lower average infection loads (S3A Table). Body metrics including median adult body size and metamorphic body mass, as well as geographic range size also appeared once each in top models for infection load. In general, larger adult body size, greater body mass at metamorphosis, and an intermediate geographic range area predicted a more intense infection intensity with Bd (S3A Table). The relationship between body metrics and infection load was driven at least partially by the fact that one of the smallest species in the study, *Pseudacris crucifer*, also exhibited the greatest resistance (lowest infection intensities) to Bd. PGLS models could explain between 13 and 37% of the variation in infection load; non-parametric GAM explained between 17 and 41% of the variation (S3A Table). Other explanatory traits appearing in candidate but not top models included lifespan and habitat breadth (S3A Table). Species with shorter lifespans and less breadth in habitat usage tended to have higher infection intensities after exposure to Bd. Assumptions of linearity were well met for habitat use metrics (breadth and central tendency) and adult body size, but non-linear relationships with infection intensity were observed for geographic range size and slightly non-linear relationships were observed for lifespan and average mass at metamorphosis (S1 Fig).

Three candidate models emerged for explaining log response ratio (LRR), or the effect size for the difference in days survived between treatments (S3B Table). From these 3 candidate models, 2 models met the criteria for both primary and secondary model selection (e.g., delta AICc ≥ 2 and an AICc scores that were smaller for the simpler/reduced model compared to the more complex models in which it was nested [40–41]). Habitat breadth and adult body size appeared as significant predictors in both top models; species lifespan and age at sexual maturity also appeared as significant predictors once each in top models (S3B Table). Species with greater breadth of habitat usage and larger body sizes who sexually mature at an earlier/younger age and have shorter lifespans also had smaller, more negative, LRR values (*e*.*g*., these species tended to experience a larger negative effect of Bd treatment on their survival). Assumptions of linearity were well-met for all explanatory variables in the LRR models. Compared to our other estimate of mortality, hazard ratio, the log response ratio models showed much greater predictive power explaining 14–35% (PGLS) and 12–33% (non-parametric GAM) of the variation (S3B Table).

The best models to explain variation in hazard ratio (HR) varied widely and were comparatively more numerous than those for infection load and log response ratios (S3C Table). In general, there was not a clear signal for any one variable to explain a great deal of variation in HR (no R^2^ value exceeded 0.23 for PGLS or 0.34 for GAM). In other words, many models were selected as “top” models according to delta AICc scores less than 2 [40], but there was not a great deal in overlap of variable importance among top models (S3 Table). Exceptions include habitat central tendency, which appeared in 4/12 candidate models for HR and adult body size, which appeared in 3/12 candidate models. In general, more ephemeral aquatic habitat use and larger body sizes were associated with increased hazard ratios. Assumptions of linearity were well-met for all explanatory variables in the HR models, except for eggs laid per year and geographic range size (S1 Fig).

Several species had such high background mortality (*e*.*g*., control mortality ≥ 30% in *Anaxyrus americanus*, *Hyla versicolor*, and *Lithobates pipiens*) that we considered them as possible outliers in our models (Fig 1). To test for the effect of these species on our comparative framework, we repeated all PGLM analyses excluding these three species. The results for infection intensity and LRR analyses were qualitatively similar to those including all species, indicating that the overall results of the phylogenetic comparative analysis were robust to these potential outliers. Model fits were significantly better and explanatory power greater for HR models when 3 species were dropped, however, perhaps indicating that these high mortality species were adding noise to this response variable. To maximize statistical power and sample sizes we emphasize analyses using all species in the main text. However we report the results excluding species with especially high mortality in our supplementary materials (S4 Table) and direct readers there for more information and comparisons to the full model analysis (S3 Table versus S4 Table).

## Discussion

Our experimental study documents a broad range of host susceptibility and vulnerability to Bd under standardized laboratory conditions, with an unprecedented evaluation across 20 North American species reared from the egg stage through metamorphosis. We observed extensive variation in host responses within and among species, and even within the same amphibian family. Here, we provide experimentally-derived estimates of survival and intensity of infection for the first time in several understudied amphibian species (S5 Table) which can be compared to what is currently known about Bd prevalence and exposure risk in the field. The spread of Bd is likely driven by both the abundance and identity of hosts in amphibian assemblages and extinctions may occur when species with high vulnerability to Bd exist in a community with less vulnerable but highly infectious hosts. Correlations among host epidemiological traits, phylogenetic history and ecological factors suggest that patterns at the supra-species level could be useful for pro-actively managing at risk species and closely monitoring the abundance and distribution of those species with the greatest competence to transmit Bd.

### Integrating Host Responses and Host Ecology to Predict Risk

Phylogenetic comparative analyses revealed patterns in host susceptibility and vulnerability to Bd. Frogs in the family *Ranidae* tended to experience lower Bd infection intensities in relation to bufonid toads and hylid chorus frogs (Fig 3A). In fact, 5 of the 7 species for which some percentage of Bd-exposed animals tested negative for infection, despite weekly experimental inoculations, were from the genus *Ranidae*. This might suggest a physiological mechanism, unique among ranids, which confers some resistance to or ability to fight Bd. For example, antimicrobial skin peptides (AMPs) in amphibian skin form a primary defense against Bd invasion of the skin [42], and a diverse array of AMPs with high inhibitory activity against Bd have been isolated from frogs in the genus *Rana* (*Lithobates*) [42–43]. A recent study showed that newly metamorphosed *Lithobates pipiens* possessed effective inhibitory skin peptides against Bd, but when these peptides were depleted from the skin, metamorphs died rapidly compared to those with peptides intact [44]. Gervasi et al. [31] showed that elevated cellular and humoral immune responses of *Rana cascadae* were associated with higher tolerance (absence of mortality during a low-dose exposure to Bd) and resistance (ability to clear Bd infection over a 15 d trial) compared to *Pseudacris regilla*. The American bullfrog (*Lithobates catesbeianus*) has been shown to persist with Bd in the wild, sometimes at low pathogen loads [24, 45] and this species is suggested to be an asymptomatic carrier or a reservoir of Bd [24]. We show here and elsewhere [38] that American bullfrogs exhibit variation in responses to Bd depending on pathogen strain but also due to individual-level heterogeneity in resistance to infection, with some individuals able to clear Bd infections in the skin, even after repeated high inoculations (S1 Table). One phylogenetic comparative study based on field detection of Bd showed that amphibians in the family Ranidae were more likely to be classified as susceptible (infection-positive versus infection-negative) than species in other amphibian families [46]. However, this classification could also result because field detection is typically biased toward sampling of live animals, who are possibly a reduced number of tolerant surviving host phenotypes. Observational and experimental accounts of susceptibility of different populations of the same species and among species in the same amphibian family can be extremely variable. In these cases, variation could be strongly driven by density-dependent host-pathogen dynamics [25] and/or rapid evolution of host resistance or tolerance to Bd.

Our phylogenetic reconstructions for hazard ratio suggest that species in the family *Bufonidae* tended to be most vulnerable to Bd-induced mortality compared with species in other amphibian families (Fig 3C) and may therefore be at greater risk of Bd-mediated population declines and extinctions when they come into contact with the pathogen. Indeed, both temperate and subtropical toad species are believed to have suffered population declines and even extinction events linked to Bd [47–50]; though, other field studies suggest that chytridiomycosis can persist as a sub-lethal chronic disease in some toad populations [51]. Mechanistically, however, it is still unclear whether some characteristics of toad skin development or composition, especially around the time of metamorphosis [29, 34–35, 37], might represent a mechanism for impaired ability to cope with Bd associated pathology. In this study, toads tended to die at a very rapid rate, often within a few days of exposure to Bd as observed elsewhere [52–53]. It is therefore possible that some species are be less tolerant of proteolytic zoospore metabolites that can cause Bd-associated pathology and physiological impairment [54], rather than the pathology associated with pathogen infection and replication, which generally takes place over a longer period of time (5–7 days). An alternative hypothesis is that Bd could potentially replicate at a faster speed on some types of amphibian skin because of variation in the ease of invasion or in structural, mechanical or chemical characteristics (*e*.*g*., skin types that enhance the frequency of initial tethering of zoospores to the skin before the process of infection into host cells begins or, skin types that are generally more permissive to the movement of small ions or particles, including zoospore metabolites that degrade skin proteins).

Ecological models revealed that habitat use was a strong predictor of average infection intensity among species, and habitat variables appeared in the majority of candidate models as a significant explanatory factor (S3 and S4 Tables). Species that occupy more ephemeral environments (such as *Hyla squirella*, *Hyla wrightorum* and *Pseudacris ornata*) tended to become more heavily infected with Bd after our experimental exposure trial, compared to hosts whose habitats are characterized by permanent water (for example, many frogs in the *Ranidae* family). More restricted habitat breadth (a tendency to use single versus multiple types of habitats ranging from ephemeral to permanently aquatic) was associated with greater survival (smaller hazard ratios and less negative/larger log response ratios), and habitat central tendency was more important in predicting hazard ratio (*e*.*g*., rate of mortality) than Bd effect size (LRR), with more ephemeral environments associated with greater risk of Bd-induced mortality. Thus, amphibian species associated with more ephemeral aquatic environments in general, tended to become more heavily infected and experienced greater mortality when they were exposed to Bd.

Ephemeral habitat associations with increased risk of infection and death are somewhat of a paradox, since Bd requires water or moist conditions to complete its life cycle and to produce new zoospores. Bd transmission may occur from host-to-host, yet most transmission is expected to occur through contact with zoospores in the aquatic environment [55]. Previous studies showed a correlation between the risk of Bd-driven population declines and association with permanent water bodies [56–57]. However, these and other studies have typically based risk on exposure probability and presence of Bd-positive samples in surviving animals in the population. Reduced frequency of exposure to Bd in the environment because of ecological affiliations with ephemeral aquatic environments may reduce contact rates, but could also mean that associated species have not evolved resistance to the pathogen, and so, accumulate high infection intensities and exhibit disproportionately high vulnerability when they are exposed to the fungus. One study with another aquatic pathogen (Ranavirus) found a similar correlation, with experimental susceptibility to a virus closely associated with host use of semi-permanent (compared to permanent) ponds [58]. Authors suggested that a high level of turnover and more ecologically dynamic semi-permanent aquatic environments could lead to intermittent exposure to the pathogen, constraining the evolution of host defenses to infection [58]. Conversely, species associated with permanent water (*e*.*g*., *Ranidae* family) typically experience more consistent environments, frequent exposure to the same pathogen, and the strongest selection pressure to evolve defenses [58]. Because Bd is a widespread generalist pathogen with an expanding range [23], anticipating pathogen impacts on species across current and future environmental ranges is critical, even if current exposure risk is low.

Mass at metamorphosis and adult body size (SVL), at the species level, were top predictors of infection intensity and vulnerability to Bd-induced mortality. In general, heavier and larger amphibian species displayed more intense infections and experienced greater impacts of Bd on their survival. A positive relationship between body size and infection intensity has been seen in a variety of host-pathogen systems [59] and also has been identified as an important predictor of infection status and vulnerability in the amphibian-Bd system [46, 57, 60]. In the case of Bd, larger body size (also correlated with greater mass) may simply represent an increased amount of keratinized epidermal tissue available for fungal colonization. We saw opposing relationships between mass and metrics of survival and infection intensity in models that took intraspecific variation into account. For example, in our experimental models, heavier individuals tended to show extended survival and lower infection intensities indicating that body mass at metamorphosis and just prior to initiation of the study might have allowed individuals to persist with and possibly resist Bd. This could occur if mass positively correlates with overall condition. However, when mass is considered a fixed species-level trait (*i*.*e*., in our PGLS and GAM models), heavier species tended to display more intense infections and larger bodied species had an increased risk of mortality. This suggests that the tendency of body mass and infection intensity to be negatively correlated within a species could be the result of infected individuals showing inhibited growth rates rather than large mass itself providing resistance.

Several other factors emerged as significant explanatory factors in models for infection intensity and survival. For example, lifespan and age at sexual maturity were a significant explanatory factor in models for survival based on log response ratio and also showed some support for hazard ratio. Species with shorter median lifespans and who mature at a younger age were the most vulnerable to the negative impacts of Bd on survival. Pace-of-life has been linked to differences in investment in immunological defenses against pathogens [61–62]. Compared to their long-lived counterparts, short-lived amphibian species might be less likely to devote resources toward specific types of pathogen defense when faced with competing demands of growth and reproduction [61–63]. Geographic range was also among the top predictors of average infection load in our among-species models, and showed a non-linear relationship with infection intensity with a peak in infection load corresponding with a range size around a median of 80 km^2^. Geographic range size may predispose some amphibian populations or species to declines, especially in the context of multiple stressors [64–65]. Larval period and number of eggs also showed some support for predictive models of vulnerability as measured by hazard ratio. We use caution in the interpretation of hazard ratio models, because this estimate of risk, essentially a ratio of the rates of mortality over differential treatment conditions, is less likely to map with phylogenetic distance. Effect size (*e*.*g*., log response ratio for the effect of Bd versus the control treatment) may be a more reliable variable for examining phylogenetically corrected predictors. We note that broadly defined “regional” effects may have also contributed to patterns in susceptibility and vulnerability that we observed, though low statistical power did not allow us to compare the effect of region (*e*.*g*., Northeast, Southeast, Northwest or Southwest) in this study.

### Contributions to Transmission

Integrating survival and infection outcomes is critical for inferring the cumulative impact a species may have on Bd dynamics. Species that persist with high levels of infection may be important in maintaining the pathogen in the environment and may have disproportionate effects on pathogen spread to other hosts [2–3]. While we did not track daily infection intensity of individuals, we did obtain estimates of infection intensity from swabs taken immediately after mortality, suggesting that we recovered a “maximum intensity” that hosts tolerated or accumulated before death. These estimates are probably high compared to zoospore loads during earlier stages of infection. However, by multiplying the average infection intensity across the experiment with the average number of days survived, we can assess the relative contribution of species in this study. For example, several species including *Pseudacris ornata*, *Hyla squirella* and *Anaxyrus boreas* could have a much greater potential for disease spread, compared to other species. The average infection load of *H*. *squirella* was approximately half of that detected in *A*. *boreas*. But because *H*. *squirella* exhibited 100% persistence with infection over the cumulative 30-day experiment, the contribution of these species to disease dynamics may be very similar. *Pseudacris ornata*, on the other hand, has a high rate of mortality after exposure to Bd, but attains such disproportionately high infection intensities, that the overall contribution during its “experimental lifetime” may exceed that of other species.

## Conclusions

In addition to host density, species composition in ecological communities can affect disease spread, in particular when some hosts have disproportionate responses to exposure and infection [3, 10, 14–15, 53]. By integrating experiments with comparative models, we show that phylogeny, ecology and intrinsic variation in responses to Bd contribute to variation in vulnerability and susceptibility to Bd. Controlled laboratory experiments such as this one provide a window into fine-scale patterns of host responses. However, we acknowledge that our standardized methods do not consider the full set of complex drivers of host responses to Bd, including differences in virulence of different Bd strains and variation in the abiotic and biotic environment [23, 66–67]. Factors such as season, climate, weather and temperature affect spatiotemporal patterns of exposure to Bd [23]. These and other factors could also interact with host physiology to drive patterns of infectiousness and mortality [67]. We emphasize the importance of considering complexity across natural landscapes, and also urge a collaborative integration of observational surveys, experimental manipulations, and semi-natural mesocom studies to continue to refine our understanding of the amphibian chytrid fungus system.

## Materials and Methods

### Collection and Husbandry

A key aspect to our study was that no hosts had previous exposure to the fungus because eggs of all species were collected from where they were laid at field locations (S6 Table) and transported to the Pymatuning Laboratory of Ecology (Crawford County, Pennsylvania) where they were reared through metamorphosis. Our rearing protocol was identical for all species across all four years of the study (2009–2012), following Searle et al. [32] and Gervasi et al. [38]. Briefly, amphibian egg masses were hatched in 200 L plastic pools containing aged well water. Free-swimming tadpoles (Gosner stage 27 [68]) were moved to 100-L plastic pools filled with 90 L of well water. After removing all aquatic predators, we combined approximately 1 L of pond water and added equal aliquots to each pool to provide a natural source of algae and zooplankton to all pools. We added 5 g of rabbit chow and 100 g of dry oak leaves (primarily *Quercus* spp.) to each pool to provide a source of nutrients and a substrate for the periphyton. Before we introduced tadpoles to the pools, the mixture sat for at least 15 d to allow the algal community to develop. Tadpole density was either 15 or 25 individuals per pool, depending on whether a species grew to moderate or large sizes. All pools were covered with cloth that provided 60% shade, excluded predators, and prevented animals from escaping. When animals reached metamorphosis (Gosner stage 42–44 [68]), we moved them from wading pools to 1-L containers lined with sphagnum moss, where they were kept until full tail absorption. We fed post-metamorphic animals pinhead crickets (*Acheta domestica*) ad libitum for 1–2 wks before shipping them overnight to the laboratory at Oregon State University (OSU), Corvallis, Oregon.

Upon arrival to OSU, we placed animals in glass terraria (51 cm X 26.5 cm X 32 cm) held at 21.5–23.3°C with 13h: 11 h light to dark photoperiod. Animals were acclimated for 24 hrs before initiation of the experiment. Our sample size for each species was N = 20 to 50 individuals (S6 Table) and we randomly assigned half of the individuals to either Bd-exposed or unexposed (control) treatments. We measured initial mass and snout-vent length of all individuals and placed them in large Petri dishes (140 X 30 mm) with holes in the lid and a thin film of water covering the bottom. We kept animals in these dishes for the duration of the experiment (30 d) and fed them twice a week with pinhead crickets at a rate that was related to the mass of the metamorphs (1 cricket per 0.1 g mass). We counted the number of crickets consumed by each frog within 24 h of feeding. Due to differences in breeding phenology and logistics, we did not test all 20 species simultaneously but instead examined species across seasons and across four years. All species were treated with identical methods in the same laboratory with the same researcher present to oversee experimental procedures (SSG).

### Ethics Statement

All methods were carried out in strict accordance with the recommendations in the Guide for the Care and Use of Laboratory Animals of the National Institutes of Health. Vertebrate animals in the study were cared for, maintained over the duration of the study, and procedures were carried out in accordance with the committee approved Institutional Animal Care and Use Committee of Oregon State University **IACUC** # 4184 and University of Pittsburgh **IACUC** #12050451. Collection of amphibian eggs for all experiments was approved by Departments of Fish and Wildlife: Oregon Scientific Taking Permits 006–09, 006–012, and 081–14 issued to A.R.B. and Pennsylvania State Collection Permit #161 issued to R.A.R. Field sampling methods for amphibian eggs included hand collecting and dip netting directly from ponds, approved in active scientific collecting permits. During experiments, animals were checked two times daily for signs of distress, including anorexia, loss of righting reflex and seizure. If signs of distress were observed, animals were euthanized by immersion in MS-222 in accordance with approved IACUC protocols. As infection and mortality due to infection were endpoints in this study, we observed mortality during the study. All infected individuals were subjected to subsequent quantitative PCR to assess infection intensity at the time of death. At the end of the 30-d experiment, all remaining animals were euthanized by immersion in MS-222 in accordance with approved IACUC protocols. No analgesics or anesthetics were used in the study but any signs of distress (above) were treated as grounds for humane euthanasia.

### Experimental Procedures

Experimental design and inoculation procedures were identical to Searle et al. [32]. All animals in the Bd treatment were exposed to 15 ml of 1.7 X 10^4^ zoospores/ml inoculate (2.6 X 10^5^ zoospores, total) during inoculations, which occurred weekly for a total of 30d. We obtained *Batrachochytrium dendrobatidis* isolate, JEL 274 (originally isolated from a Western toad in Colorado, USA [69]) as a cryogenically preserved culture from Joyce Longcore, University of Maine. Live Bd cultures growing on 1% tryptone agar plates were harvested into liquid culture (1% tryptone broth) and re-cultured every 2–3 months. For years 2–4 of the study, we obtained new cryogenically preserved cultures of the same stock strain from J. Longcore and repeated the process of culturing before experiments. For year 1 of the study, new cryogenically preserved culture was obtained within 1 year prior to initiation of the study.

For all experimental inoculations, Bd was cultured from liquid broth to 100 mm X 15 mm tryptone agar plates and allowed to grow for 6 d at 23°C before inoculation of animals. Previous studies have shown that zoospore activity and density with this particular strain of Bd are highest within 6–8 d after culturing the pathogen on tryptone agar plates [30–32, 38]. Zoospores were harvested by flooding agar plates with 10 ml of dechlorinated water and then scraping the surface of the agar. We pooled the inoculums of several (5–10) plates. All zoospore counts were determined by hemocytometer from pooled inoculation broth. Zoospores were transferred to individual petri dishes, already containing 10 ml of dechlorinated water. Control animals were exposed to the same volume of sham inoculation (created from pathogen-free tryptone-agar plates). Thus, throughout the experiment, a thin layer of inoculated water was present at the bottom of the petri dish. Animals were therefore exposed to, but not immersed in, the diluted inoculate and all species were observed to be able to move across the bottom and sides of the petri dishes (Gervasi personal observation). Our inoculation method standardized pathogen exposure regimes among amphibian species, allowing us to assess baseline differences in species responses to the same treatment regime as we have done previously [30–32, 38].

For all species, metamorphic animals of the same age were randomly assigned to one of two treatments (Bd-exposed or sham inoculate). In most cases, total sample size was 50 animals, with half of the animals receiving each treatment. However, sample sizes varied depending on egg availability and survival to metamorphosis (S6 Table). Water was changed concurrent with re-inoculation every 7 d. Animals that died during the experiment were immediately preserved in 95% ethanol. Animals remaining alive at the end of the 30 d experiment were euthanized in accordance with institutional animal care protocol in MS-222 and then preserved in 95% ethanol.

We used quantitative-PCR (qPCR) to assess infection load in the skin of post-metamorphic amphibians [70]. We performed qPCR to quantify infection load following the methods of Boyle et al. [70], except that we used 60 ul of Prepman Ultra (Applied Biosystems, Grand Island, NY) instead of 40 ml in all DNA extractions. Extractions were diluted 1:10 and processed in an ABI PRISM 7500 (Applied Biosystems, Grand Island, NY). Each sample was analyzed in triplicate and the average number of genome equivalents of Bd per animal was calculated. Ventral abdominal skin and inner thigh skin of preserved animals was swabbed for Bd using fine tipped sterile swabs (Medical Wire and Equipment MW&E 113, Wiltshire, England) for Bd. We sampled all Bd-exposed animals of each species at death or at the end of the experiment (d 30) if they survived, and also randomly sampled at least 5 control animals of each species to ensure no contamination across treatments. Control animals were sampled randomly and included both animals that died during the experiment as well as those that survived the entire experimental duration.

### Statistical Analyses

We performed statistical analyses in R 3.2.3 [71]. We created Kaplan-Meier survival curves to display the differences in the rate of mortality between the control and Bd treatments for each species. To statistically compare mortality rate within and among species, we used a Cox’s proportional hazards model ([33], “coxph” function in the R package “survival” [72]), which is a non-parametric regression model. A hazard ratio (and associated p-value for the effect being modeled) is a useful comparative indicator of the risk among species because it is calculated based on mortality in both the control and the Bd treatment (e.g., it is relativized). A hazard ratio > 1 indicates an increase in the probability of mortality and a hazard ratio = 1 indicates equal mortality between or among 2 or more groups. Within species models included treatment as a main effect (and no-covariates); among-species Cox models were used to examine how multiple factors (*e*.*g*., species ID, treatment, mass) influenced survival in the experiment. In our among-species model for survival, we initially examined all possible candidate Cox regression models that included both species identity and treatment as main effects (i.e., we made the *a priori* decision that these two factors were biologically relevant based on our questions of interest about species and treatment effects). We also included mass as a possible factor in survival models that took individual and species-level variation into account. We tested all possible single factor and 2-way interactions between species identity, treatment, and mass as explanatory factors for survival. We also only chose as possible models those that included interactions where the main effects were also present in the model (10 possible models, total). We considered as candidate models those with delta AICc values less than or equal to 2 according to Burnham and Anderson's rule of thumb [40] and also removed more complex models from this candidate list that did not have a delta value lower than the simpler models within which they were nested [41]. We present only the final top model in our results section. Results for main and interaction model effects are summarized using Wald chi-square (type II) tests (“Anova” function in the R package “car” [73]).We used the package “AICcmodavg” to compare AICc scores and delta AICc values among all candidate models [74]. In addition to hazard ratios, we also calculated the log response ratio (*e*.*g*., effect size) for the difference between number of days to death in the control and Bd-exposed treatments. Log response ratio was used as a secondary “survival” response variable in the fitting of ecological models with experimental data, since it represents a different view of the survival effect (*e*.*g*., effect size for difference in average number of days survived between the Bd and control treatment). Log response ratio was calculated according to the method of Lajeunesse (2011, [75]) as: Ln (Xt / Xc); where Ln equals the natural log, Xt = the average number of days survived in the Bd treatment group, and Xc is the average number of days survived in the control group. Thus, smaller, more negative values of LRR are associated with a larger negative Bd effect on survival.

For infection-load analyses, we transformed quantitative-PCR loads (log (raw genome equivalents per individual + 1)) to normalize data and used a linear model examine among- species differences in infection load across species. We examined the predictive power of species identity, number of days survived, and mass in our linear model We decided *a priori* to choose a model that included species identity as a main effect because our study was designed specifically to examine species-level variation. We considered all single factor and 2-way interactions between explanatory variables for infection intensity (13 possible models). We present the results from the final top model selected in our results section.

We examined unequal numbers of species within each of the four years. We did not test the same species in more than a single year (S6 Table). Within each year (with the exception of year 4, where n = 1), the conclusions of our analyses (e.g., significant differences in survival and infection responses at the species level) were in agreement with conclusions when years were pooled. Experimental results from 7 of 20 species presented in our analysis are included from previous work by the authors [32, 38]. Though survival curves are reproduced nearly identically, our within-species hazard ratios calculated for the Bd treatment effect presented in this study were obtained from Cox Proportional Hazards models that were fit without covariates. This explains why exact values differ from those previously reported [32, 38].

### Species Attributes and Ecological Traits

We collected information on species attributes (S7 Table) by searching species accounts from two amphibian references [76–77] and four global databases [78–81]. For a single species (*H*. *wrightorum*), information on eggs per year, age at maturity, and larval period was not available in any of the above cited literature, and estimates for these traits were obtained from field investigators who collected eggs of this species for the study (J. Collins and O. Hyman, *personal communication*). For all trait estimates, we gathered the entire range of the metric as reported among the various sources where we located species-specific information. We obtained the median value of this range for use in parameterizing our models (S7 Table). We obtained geographic range size estimates for our 20 species of interest from a subset list of species included in Lawler et al. [82]. Average metamorphic body size of animals at the start of our experiment was used as an additional ecological species attribute.

### Species Habitat Affiliation

To categorize species’ habitat use and habitat breadth, we used the published protocol of Van Buskirk [83] by generating a list of scientists that have published at least one paper on a species included in our study within the last 15 years using Web of Science. Each scientist was sent a survey (190 scientists in total) and asked to rank each species’ habitat usage on the following scale: 1) “common”, 2) “uncommon”, 3) “rare”, or 4) “never” observed across each of the five habitat types (S7 Table). The five habitat types were as follows: (1) dries within weeks with no or few small invertebrate predators, (2) dries every year with few small invertebrate predators, (3) dries every few years and occasionally has large invertebrate predators, (4) never dries and has large invertebrate predators, and (5) never dries and has fish and large invertebrate predators. We received completed surveys from 56 scientists with 323 total responses across all the species included in this study. One species (*H*. *wrightorum*) only had one response; all other species had at least 5 responses. We converted the categorical rankings of never, rare, uncommon, and common into three different numerical estimates to fully explore the potential variation found in habitat use or habitat central tendency (S7 Table).

The first habitat estimate (henceforth the doubling system) used a weighting system where never was zero, rare was one, uncommon was two, and common was four. The second habitat estimate (henceforth the squaring system) used a weighting system where never was again zero, rare was again one, uncommon was four, and common was sixteen. The third habitat estimate (henceforth the exponential system) used a weighting system where never again was zero, rare was again one, uncommon was ten, and common was one hundred. We then converted our five habitats into numbers starting with the shortest hydro-period as one and the longest hydro-period with fish predators as five. We then summed the numbers within each habitat type across all the scientists’ responses and checked what the average response was within each habitat. If a species averaged less than rare in a habitat, it was eliminated and that species usage of that habitat was set to zero to reflect the fact that habitats used so infrequently are likely of limited evolutionary importance to a species. We next converted those sums into proportions for each habitat and then weighted those proportions by multiple each proportion by the habitat type number. This generated a continuous number between one and five that estimates the average habitat use for each species. All species except one (*A*. *boreas*) produced a unimodal distributions and even the *A*. *boreas* was unimodel for one of the three estimates (the doubling system). For the other two estimates the distribution was bi-modal, but with a very flat set of peaks with little difference between the three middle habitat types (the trough was two and three percent lower than the lower peak).

Lastly, we estimated habitat breadth by assaying the spread of each distribution (S7 Table). From the average habitat use, we measured how far into each adjoining habitat or habitats did one have to go to account for forty percent of the distribution in each direction (in total eighty percent of the distribution centered on the average). This produced an estimate that ranged from zero if the species was only found in one habitat to three when a species was found in all five habitats equally. For *A*. *boreas*, regardless of the weighting system the distribution was wide enough to span from habitat one through habitat five, thus each estimate ended in a habitat type that did not include a peak.

### Phylogenetic Comparative Analyses

Phylogenetic signal can be defined as a pattern of trait variation whereby more closely related species have more similar traits (*i*.*e*., trait disparity among species is correlated with the phylogenetic distance that separates them). We tested for phylogenetic signal in host responses to Bd and our explanatory variables (*i*.*e*., species habitat affiliations, ecological associations and life history traits) using a standard measure of phylogenetic signal: Blomberg’s K [84]. Blomberg’s K varies between zero and ∞, with values of zero indicating phylogenetic independence, values <1 indicating partial phylogenetic dependence (i.e., weaker signal) than expected under a Brownian motion model of character evolution, and values >1 indicating greater phylogenetic dependence than expected under Brownian motion. Blomberg’s K was calculated for all three measures of susceptibility to chytrid fungus (our response variables) as well as all predictor variables used to build models of susceptibility. Evolutionary trends among response variables were visualized using maximum likelihood ancestral character reconstruction [85] with the software package Picante v1.6–2 [86], implemented in R v3.0.1. Two phylogenetic trees were used to quantify phylogenetic distance among species (Table 1). One tree was based on previous studies of anuran phylogeny and assumed a speciational model of trait evolution (*i*.*e*., all branch lengths were set to one), and the other tree was estimated via maximum likelihood from 2500 bp of mitochondrial sequence data obtained from GenBank. Further details of the methods used to create these two trees are detailed in Table 1. The statistical significance of observed signal was assessed using a randomization procedure [87] and 1000 permutations per trait. Evolutionary trends among response variables were visualized using maximum likelihood ancestral character reconstruction [85]), implemented in the R package *ape* v 3.1–4 [88]. We used phylogenetic least squares (PGLS) implemented using the R package Caper v. 0.5 [89] to construct models of response variables [89], and additionally tested for non-linear relationships among variables with generalized additive models (GAM) implemented in mgcv v 1.7–28 [90].

Preliminary analyses showed that some of the response variables and many of the predictor variables showed weak but statistically significant phylogenetic signal. We therefore initially used a phylogenetically informed method, phylogenetic generalized least squares (PGLS, [91]), to construct models of response variables. This method was implemented using the R package Caper v. 0.5 [89]. *A priori* we had identified a number of variables that might explain variation in the response variables, but lacked evidence as to which would be most important or even if all of the explanatory variables would be correlated with all of the response variables. Therefore, for each response variable, we compared the AICc scores (using AICc rather than AIC scores to correct for the small sample sizes from which our models were constructed), of models that included every possible combination of response variables. When fitting our PGLS models we used a maximum likelihood estimate of lambda [92], a measure of phylogenetic signal similar to Blomberg’s K, to determine the amount of phylogenetic correction to be applied to variables in the model. Somewhat surprisingly, the maximum likelihood value of lambda estimated by Caper invariably proved to be zero, indicating that no phylogenetic correction was applied to variables included in a given model (S2 Table). This generally indicates a lack of phylogenetic signal in the residual error of a PGLS model [39]. Further exploratory analyses confirmed that forcing the inclusion of phylogenetic correction by manually setting lambda values other than zero hurt overall model fits and result in inflated AICc scores.

One limitation of the PGLS method is that it assumes that linear relationships between predictor and response variables. To determine the direction of relationships between predictor and response variables and to test for possible nonlinear relationships between predictor and response variables we reconstructed all of our best fitting models, defined as the set of models with AICc scores that differed by less than two from that of the model with the lowest AICc score for a given response variable using generalized additive models (GAM) implemented in mgcv v 1.7–28 [90]. In basic terms, a GAM is a type of generalized linear model that can detect non-parametric relationships by incorporating smoothing functions [93–95]. Each smoothing function allows for some departure from strict linearity, and in mgcv model fits are evaluated using penalized likelihood so that the simplest model that is a reasonable fit to the data is preferred [94]. With the exception of geographic range area and lifespan, these analyses confirmed that the relationship between predictor and response variables were linear, and thus that the assumptions of the PGLS analysis had been met, with two notable exceptions (S1 Fig).

## Supporting Information

S1 Table Summary of species traits and amphibian responses to *Batrachochytrium dendrobatidis*. (PDF)

S2 Table Example of lambda values for 10 phylogenetic least squares models for average infection load. (PDF)

S3 Table Best predictive models for species-level traits explaining (A) infection load (IL), (B) log response ratio (LRR) and (C) hazard ratio (HR).
(PDF)

S4 Table Re-fit predictive PGLS models for (A) infection load (IL), (B) log response ratio (LRR) and hazard ratio (HR) with 3 amphibian species removed (i.e., those with high control mortality including *L*. *pipiens*, *H*. *versicolor*, and *A*. *americanus*).
(PDF)

S5 Table Known sampling coverage by country and state, with Bd detections for species in this study. (PDF)

S6 Table Amphibian species information, egg collection location, sample sizes and year of study. (PDF)

S7 Table Ecological and life-history summaries for species in experimental study. (PDF)

S1 Fig Non-linear relationships from GAM analysis. (PDF)
