# Supplementary material for: Linking Ecology and Epidemiology to Understand Predictors of Multi-Host Responses to an Emerging Pathogen, the Amphibian Chytrid Fungus
Source: PLoS One. 2017 Jan 17;12(1):e0167882. doi: 10.1371/journal.pone.0167882 (PMC5240985; doi:10.1371/journal.pone.0167882)
Supplement: S1 Table — (PDF) [file pone.0167882.s001.pdf]

S1 Table S1. Summary of species traits and amphibian responses to *Batrachochytrium dendrobatidis*.

Genus abbreviations: A = *Anaxyrus*; H = *Hyla*; P = *Pseudacris*; R = *Rana*; L = *Lithobates*. Other abbreviations: Ave = Average, SVL = snout-vent-length. Sample sizes for each species are provided in Table S7. Hazard ratios presented in column 4 were obtained from Cox Proportional Hazards models that were fit without covariates, which explains why exact values differ from those previously reported [32, 38]. Log response ratio in column 5 denotes the effect size for differences in average number of days survived between the two treatments, Bd-exposed and Control, and was calculated as Ln (Xt / Xc), where Ln equals the natural log, Xt = the average number of days survived in the Bd treatment group, and Xc is the average number of days survived in the control group. Smaller log response ratio values indicate a larger negative effect of Bd on amphibian survival. Average infection intensity in column 7 was calculated across the entire 30 d experiment for all Bd-exposed animals. All infection intensity estimates are provided as raw (untransformed) genome equivalent values from quantitative-PCR analysis. Average infection intensities recovered from amphibians who died versus those who survived are provided in column 8; n/a indicates that no individuals survived to the end of the experiment, and only average infection load values (column 7) are applicable. For column 4 and 6, n/a indicates no HR calculated due to data distribution precluding analysis and absence of mortality for *A. terrestris* and *H. squirella*, respectively.

Species	Ave mass	Ave SVL	Hazard ratio for treatment effect	Log response ratio for treatment effect	Day @ 50% mortality in Bd-exposed	Ave infection load (30d total)	Ave infection load of individuals that died versus survived in experiment	% of animals survived in Bd-treatment	% of Bd-exposed individuals that were Bd-negative	Infection load coefficient of variation	Range of infection load values (min versus max detected)
A. americanus	0.20	12.33	48.3	-1.231	3	206.19	n/a	0	0	1.56	15.0 - 1630
A. boreas	0.21	11.87	17.7	-0.783	12	1473.41	n/a	0	0	1.29	58.3 - 8415
A. fowleri	0.28*	14.21	37.4	-1.508	5	313.38	n/a	0	0	1.19	4.69 -1239
A. terrestris	0.15	11.45	n/a	-2.014	3	174.05	n/a	0	0	0.90	1.91 - 545
H. squirella	0.25	14.56	1	undefined	n/a	710.75	n/a	100	0	1.69	1.59 - 3510
H. versicolor	0.27^	14.61	2.83	-0.978	2	57.35	n/a	0	0	1.27	0.59 - 335
H. wrightorum	0.38	14.95	22	-0.244	24.5	357.08	570.52 / 58.26	42	0	1.81	3.54 - 2690
L. catesbeianus	1.31*^	24.95	11.4	-0.957	5	43.1	56.25 / 0.35	24	12	1.04	0 - 141
L. clamitans	1.13^	20.98	3.34	-0.805	4.5	112.89	138.59 / 10.06	20	0	2.22	1.95 - 819
L. pipiens	1.00*^	23.39	5.51	-1.302	2	98.59	102.68 / 0.283	4	0	0.92	0.09 - 329
L. sphenocephalus	0.81*	20.82	65.2	-1.450	3	358.33	389.49 / 0	8	8	0.78	0 - 1029
L. sylvaticus	0.30*^	15.16	20	-2.097	2	133.25	n/a	0	0	1.12	8.41 - 616
P. crucifer	0.16*	12.78	4.59	-0.471	15	1.41	1.53 / 0.007	8	4	1.11	0 - 5.35
P feriarum	0.20	13.14	4.7	-1.063	5	281.32	n/a	0	0	0.97	12.3 - 954

P. ornata	0.46	15.60	41.6	-0.930	11	4530.42	n/a	0	0	0.81	678 - 12029
P. regilla	0.30	14.13	37.5	-1.669	3	118.86	n/a	0	0	2.14	1.56 - 983
P. triseriata	0.17*^	13.20	16.9	-0.592	16	72.63	82.34 / 1.39	12	8	1.61	0 - 396
R. aurora	0.62	18.02	4.72	-0.938	3	49.7	64.13 / 0.61	23	4.5	1.58	0 - 292
R. cascadae	0.46*	16.05	5.35	-0.623	7	66.79	82.21 / 0	19	19	1.93	0 - 457
R. luteiventris	0.58^	18.30	5.43	-0.608	19	121.21	145.27 / 0.72	17	4	1.98	0 - 1070

Asterisks signs (*****) next to average mass values indicate species for which the effect of mass on days survived was significant at P = 0.05 in a Cox regression model. In all cases, greater mass was associated with longer survival times. Caret signs (**^**) next to average mass values indicate species for which the effect of mass on infection intensity was significant at P = 0.05 in a linear regression model. In all of these species except one (*H. versicolor*) larger starting body mass was associated with lower average experimental infection intensities/infection loads.
