# Supplementary material for: Linking Ecology and Epidemiology to Understand Predictors of Multi-Host Responses to an Emerging Pathogen, the Amphibian Chytrid Fungus
Source: PLoS One. 2017 Jan 17;12(1):e0167882. doi: 10.1371/journal.pone.0167882 (PMC5240985; doi:10.1371/journal.pone.0167882)
Supplement: S2 Table — (PDF) [file pone.0167882.s002.pdf]

**S2 Table. Example test of lambda values for 10 phylogenetic least squares models for average infection load.** As phylogenetic signal is reduced, model fit improves as measured by AICc values. This same trend was observed for the two additional response variables, log response ratio (LRR) and hazard ratio (HR). Thus, all PGLS models were performed with reduced lambda values. AICc scores marked with identical superscripts (^, *, or #) represent identical models, showing the trend for better model fit with reduced values of lambda.

| λ=0.5 | AICc | λ=0.1 | AICc | λ=0.001 | AICc | λ=0.0001 | AICc | λ=0.00001 | AICc |
|---|---|---|---|---|---|---|---|---|---|
| | 74.64^ | | 73.95* | | 72.77* | | 72.76* | | 72.76* |
| | 74.68 | | 74.12^ | | 73.31# | | 73.30# | | 73.30# |
| | 75.44 | | 74.13# | | 73.71^ | | 73.70^ | | 73.70^ |
| | 75.71 | | 74.25 | | 73.87 | | 73.87 | | 73.87 |
| | 76.05# | | 74.90 | | 74.05 | | 74.04 | | 74.04 |
| | 76.79 | | 75.30 | | 74.20 | | 74.19 | | 74.19 |
| | 77.00* | | 75.54 | | 74.50 | | 74.48 | | 74.48 |
| | 77.09 | | 75.80 | | 74.99 | | 74.98 | | 74.98 |
| | 77.49 | | 76.17 | | 75.49 | | 75.48 | | 75.48 |
| | 77.50 | | 76.21 | | 75.99 | | 75.99 | | 75.99 |
