# Supplementary material for: Linking Ecology and Epidemiology to Understand Predictors of Multi-Host Responses to an Emerging Pathogen, the Amphibian Chytrid Fungus
Source: PLoS One. 2017 Jan 17;12(1):e0167882. doi: 10.1371/journal.pone.0167882 (PMC5240985; doi:10.1371/journal.pone.0167882)
Supplement: S3 Table — Best predictive models for species-level traits explaining (A) infection load (IL), (B) log response ratio (LRR) and (C) hazard ratio (HR). (PDF) [file pone.0167882.s003.pdf]

S3 Table. Best predictive models for species-level traits explaining (A) infection load (IL), (B) log response ratio (LRR), and (C) hazard ratio (HR). Phylogenetic least squares (PGLS) models were run with lambda set to 0.00001. Comparison of linear (PGLS) and non-parametric generalized additive models (GAMs) is made with adjusted R^2 values. GAMs allowed us to assess the presence of non-linear associations between species traits and responses to Bd and also allowed us to interpret the direction of associations. Candidate models, chosen based on delta AICc scores less than 2, are listed for each response variable. From these candidate models, top models (highlighted in yellow) were selected by excluding any model that contained repeated variables and additional terms without improving model fit. For each response variable, we examined 129 possible combinations of 9 explanatory factors (single factor up to 9 factor models). No interactions were considered between species-level explanatory variables. Conclusions are drawn for each response variable by listing the identity and frequency of each explanatory trait in candidate and top models. An asterisk is given where the effect of that explanatory trait was significant at p = 0.05 or less. A degree symbol (°) denotes model traits with p values equal between 0.051 and 0.080 (“suggestive” but not significant).

Explanatory trait key

1. Habitat central tendency (HCT): an estimate of habitat use scaled from 1 (ephemeral; insect predators) to 5 (permanent; vertebrate predators).

2. Habitat breadth (HB): an estimate of habitat use scaled from 1 (only 1 type of habitat occupied ranging from ephemeral to permanent) to 3 (exists in all habitats; exhibits usage of a range of habitat types).

3. Adult body size (ABS): median adult body size (mm)

4. Average body mass (ABM): median body mass at metamorphosis (g)

5. Geographic range area (GRA): median geographic range area for the species (km^2)

6. Eggs per year (EPY): median number of eggs laid by females of the species in a year

7. Lifespan (LS): median lifespan (years)

8. Larval period (LP): median larval period (weeks)

9. Age at sexual maturity (ASM): median age at sexual maturity (months)

A. Average Infection Load (IL)

AICc Score	Model Composition	PGLS adjusted r-squared	PGLS model p-value	GAM adjusted r-squared
75.33	IL ~ HCT* + ABS*	0.241	0.037	0.352
75.40	IL ~ ABM + HCT* + ABS°	0.309	0.030	0.311
75.92	IL ~ HCT* + HB + ABS* + LS°	0.369	0.026	0.411
76.41	IL ~ GRA°	0.130	0.066	0.198
76.54	IL ~ HCT* + HB + ABS*	0.269	0.046	0.268
77.06	IL ~ ABM + HCT*	0.172	0.078	0.174

Infection Load Summary:

(1) **Habitat central tendency** (HCT) in 5/6 candidate models and 2/3 top models. Relationship with infection load is negative and linear.

(2) **Adult body size** (ABS) in 4/6 candidate models and 1/3 top models. Relationship with infection load is positive and linear.

(3) **Geographic range area** (GRA) in 1/6 candidate models and 1/3 top models. Relationship with infection load is negative and non-linear (hump shaped).

(4) **Average body mass** (ABM) in 2/6 candidate models and 1/3 top models. Relationship with infection load is positive and non-linear (positive and then plateau).

(5) **Habitat breadth** (HB) in 2/6 candidate models. Relationship with infection load is negative and linear.

(6) **Lifespan** (LS) in 1/6 candidate models. Relationship with infection load is negative and slightly non-linear.

B. Log response ratio (LRR)

AICc Score	Model Composition	PGLS adjusted r-squared	PGLS model p-value	GAM adjusted r-squared
26.71	LRR ~ HB* + ABS* + ASM*	0.350	0.024	0.332
27.96	LRR ~ HB* + ABS* + LS*	0.306	0.037	0.234
28.26	LRR ~ HB°	0.141	0.063	0.123

Log Response Ratio Summary:

(1) **Habitat breadth** (HB) in 3/3 candidate models and 2/3 top models. Relationship with response ratio is negative and linear.

(2) **Adult body size** (ABS) in 2/3 candidate models and 2/3 top models. Relationship with response ratio is negative and linear.

(3) **Age at sexual maturity** (ASM) in 1/3 candidate models and 1/3 top models. Relationship with response ratio is positive and linear.

(4) **Lifespan** (LS) in 1/3 candidate models and 1/3 top models. Relationship with response ratio is positive and linear.

Recall that log response ratio values are smaller (more negative) if the difference in average survival between treatments is larger. Conversely, log response ratio values are larger (less negative) if the treatment effect on survival is smaller.

C. <u>Hazard Ratio (HR)</u>

AICc Score	Model Composition	PGLS adjusted r-squared	PGLS model p-value	GAM adjusted r-squared
83.91	HR ~ HCT	0.006	0.308	-0.002
84.11	HR ~ HCT° + ABS	0.083	0.198	0.068
84.23	HR ~ LP	-0.011	0.383	-0.006
84.25	HR ~ HB	-0.011	0.387	0.150
84.28	HR ~ LS	-0.014	0.396	0.006
84.65	HR ~ ASM	-0.004	0.529	-0.025
85.01	HR ~ ABS	-0.053	0.771	-0.054
85.09	HR ~ ABM	-0.058	0.900	-0.058
85.10	HR ~ EPY	-0.058	0.942	0.121
85.11	HR ~ GRA	-0.059	0.993	-0.06
85.26	HR ~ HCT + EPY	-0.003	0.400	0.138
85.79	HR ~ HCT* + HB + ABS* + LS*	0.229	0.106	0.345

Hazard Ratio Summary:

(1) **Habitat central tendency** (HCT) in 4/12 candidate models and 1/9 top models. Relationship with hazard ratio is negative and linear.

(2) **Adult body size** (ABS) in 3/12 candidate models and 1/9 top models. Relationship with hazard ratio is positive and linear.

(3) **Larval period** (LP) in 1/12 candidate models and 1/9 top models. Relationship with hazard ratio is negative and linear.

(4) **Habitat breadth** (HB) in 2/12 candidate models and 1/9 top models. Relationship with hazard ratio is positive and non-linear (slightly U-shaped).

(5) **Lifespan** (LS) in 2/12 candidate models and 1/9 top models. Relationship with hazard ratio is negative and linear.

(6) **Age at sexual maturity** (ASM) in 1/12 candidate models and 1/9 top models. Relationship with hazard ratio is negative and linear.

(7) **Average body mass** (ABM) in 1/12 candidate models and 1/9 top models. Relationship with hazard ratio is negative and linear, but extremely shallow.

(8) **Eggs laid per year** (EPY) in 2/12 candidate models and 1/9 top models. Relationship with hazard ratio is positive and non-linear (shallow and U-shaped).

(9) **Geographic range area** (GRA) in 1/12 candidate models and 1/9 top models. Relationship with hazard ratio is negative and linear, but extremely shallow.
