# Supplementary material for: Linking Ecology and Epidemiology to Understand Predictors of Multi-Host Responses to an Emerging Pathogen, the Amphibian Chytrid Fungus
Source: PLoS One. 2017 Jan 17;12(1):e0167882. doi: 10.1371/journal.pone.0167882 (PMC5240985; doi:10.1371/journal.pone.0167882)
Supplement: S4 Table — Re-fit predictive PGLS models for (A) infection load (IL), (B) log response ratio (LRR) and hazard ratio (HR) with 3 amphibian species removed (i.e., those with high control mortality including L. pipiens, H. versicolor, and A. americanus). (PDF) [file pone.0167882.s004.pdf]

**S4 Table. Re-fit predictive PGLS models for (A) infection load (IL), (B) log response ratio (LRR) and hazard ratio (HR) with 3 amphibian species removed (e.g., those with high control mortality including *L. pipiens*, *H. versicolor*, and *A. americanus*.** Phylogenetic least squares (PGLS) models were run with lambda set to 0.00001. Candidate models, chosen based on delta AICc scores less than 2, are listed for each response variable. From these candidate models, top models (highlighted in yellow) were selected by excluding any model that contained repeated variables and additional terms without improving model fit. For each response variable, we examined 129 possible combinations of 9 explanatory factors (single factor up to 9 factor models). No interactions were considered between species-level explanatory variables. Conclusions are drawn for each response variable by listing the identity and frequency of each explanatory trait in candidate and top models. We summarize differences in model selection between the full and reduced species datasets in column 5 of each panel (A-C).

## Explanatory trait key

**1. Habitat central tendency (HCT)**: an estimate of habitat use scaled from 1 (ephemeral; insect predators) to 5 (permanent; vertebrate predators).

**2. Habitat breadth (HB)**: an estimate of habitat use scaled from 1 (only 1 type of habitat occupied ranging from ephemeral to permanent) to 3 (exists in all habitats; exhibits usage of a range of habitat types).

**3. Adult body size (ABS)**: median adult body size (mm)

**4. Average body mass (ABM)**: median body mass at metamorphosis (g)

**5. Geographic range area (GRA)**: median geographic range area for the species ($km^2$)

**6. Eggs per year (EPY)**: median number of eggs laid by females of the species in a year

**7. Lifespan (LS):** median lifespan (years)

**8. Larval period (LP)**: median larval period (weeks)

**9. Age at sexual maturity (ASM)**: median age at sexual maturity (months)

## A. Reduced – Infection Load (IL)

<table>
<tr><th>AICc Score</th><th>Model Composition</th><th>PGLS adjusted r-squared</th><th>PGLS model p-value</th><th rowspan="5">New candidate and top factors in reduced: ASM<br><br>Missing candidate and top factors in reduced: LS, HB</th></tr>
<tr><td>66.72</td><td>IL ~ HCT* + ABS*</td><td>0.270</td><td>0.043</td></tr>
<tr><td>67.09</td><td>IL ~ GRA°</td><td>0.170</td><td>0.056</td></tr>
<tr><td>68.02</td><td>IL ~ ABM + HCT* + ABS°</td><td>0.308</td><td>0.051</td></tr>
<tr><td>68.64</td><td>IL ~ ASM + GRA°</td><td>0.182</td><td>0.096</td></tr>
</table>

**Reduced – Infection Load Summary:**

(1) **Habitat central tendency** (HCT) in 2/4 candidate models and 1/2 top models.

(2) **Adult body size** (ABS) in 2/4 candidate models and 1/2 top models.

(3) **Geographic range area** (GRA) in 2/4 candidate models and 1/2 top models.

(4) **Average body mass** (ABM) in 1/4 candidate models.

(5) **Age at sexual maturity** (ASM) in 1/4 candidate models.

## B. Reduced - Log Response Ratio (LRR)

| AICc Score | Model Composition | PGLS adjusted r-squared | PGLS model p-value | **New candidate and top factors in reduced:**<br>GRA<br>EPY<br><br>**Missing candidate and top factors in reduced:**<br>none |
|---|---|---|---|---|
| 26.79 | LRR ~ HB | 0.149 | 0.077 | |
| 27.44 | LRR ~ HB* + ABS* + ASM° | 0.321 | 0.055 | |
| 27.74 | LRR ~ HB° + ABS° + LS° | 0.308 | 0.061 | |
| 28.45 | LRR ~ LS° + EPY* + GRA* | 0.277 | 0.078 | |
| 28.61 | LRR ~ EPY | 0.047 | 0.208 | |

**Reduced - Log Response Ratio Summary:**

(1) **Habitat breadth** (HB) in 3/5 candidate models and 1/2 top models.

(2) **Lifespan** (LS) in 2/5 candidate models and 1/2 top models.

(3) **Geographic range area** (GRA) in 1/5 candidate models and 1/2 top models.

(4) **Eggs laid per year** (EPY) in 2/5 candidate models and 1/2 top models.

(5) **Adult body size** (ABS) in 2/5 candidate models.

(6) **Age at sexual maturity** (ASM) in 1/5 candidate models.

## C. Reduced - Hazard Ratio (HR)

| AICc Score | Model Composition | PGLS adjusted r-squared | PGLS model p-value | **New candidate and top factors in reduced:** |
|---|---|---|---|---|
| 67.66 | HR ~ ABS° + ASM* | 0.230 | 0.072 | none |
| 68.48 | HR ~ ASM | 0.089 | 0.140 | **Missing candidate and top factors in reduced:** |
| 68.59 | HR ~ ABS* + LP + ASM* | 0.296 | 0.067 | |
| 69.67 | HR ~ HB + ABS° +ASM* | 0.247 | 0.097 | HCT<br>EPY<br>GRA<br>LS<br>ABM |

**Reduced - Hazard Ratio Summary:**

(1) **Adult body size** (ABS) in 3/4 candidate models and 1/1 top model.

(2) **Age at sexual maturity** (ASM) in 4/4 candidate models and 1/1 top model.

(3) **Larval period** (LP) in 1/4 candidate models.

(4) **Habitat breadth** (HB) in 1/4 candidate models.
