# Supplementary material for: Linking Ecology and Epidemiology to Understand Predictors of Multi-Host Responses to an Emerging Pathogen, the Amphibian Chytrid Fungus
Source: PLoS One. 2017 Jan 17;12(1):e0167882. doi: 10.1371/journal.pone.0167882 (PMC5240985; doi:10.1371/journal.pone.0167882)
Supplement: S5 Table — (PDF) [file pone.0167882.s005.pdf]

**S5 Table**. Known sampling coverage by country and state, with Bd detections for species in this study. Note: Because Bd was not detected in a particular species at a site does not imply that Bd was not found at that site in another species, or that the site is "negative" for Bd. Numbers are for detections in the particular species; more than one species may have been sampled at the same site.

| | | Sites where Bd was sampled | | | | Countries sampled[1] | | States/Provinces sampled[2] | | | |
|---|---|---|---|---|---|---|---|---|---|---|---|
| **Species Name** | Total | # sites detected in sp. | % sites detected in sp. | # sites where not detected in sp. | % sites not detected in sp. | w/sites where detected in sp. | w/sites where not detected in sp. | w/sites where detected in sp. | w/sites where not detected in sp. | State eggs collected | Bd detection (sites per species) in egg-collection state |
| *Hyla squirella* | 5 | 0 | 0 | 5 | 100 | none | US | none | FL, LA, VA | FL | not detected in 2 sites in FL |
| *Hyla wrightorum* | 1 | 1 | 100 | 0 | 0 | US | none | AZ | none | AZ | detected in 1 site in AZ |
| *Hyla versicolor* | 23 | 2 | 9 | 21 | 91 | CA, US | US | QC, CT | CT, LA, MA, ME, MN, MO, OK, PA, TX, VA | PA | not detected in 1 site in PA |
| *Pseudacris regilla*[3] | 223 | 84 | 38 | 139 | 62 | US | US, MX | CA, NV, OR, WA | CA, OR | OR | detected in 1/16 sites in Oregon |
| *Pseudacris ornata* | 1 | 0 | 0 | 1 | 100 | none | US | none | GA | FL | not previously sampled in FL |
| *Pseudacris triseriata* | 53 | 20 | 38 | 33 | 62 | CA, US | US | QC, AZ, CO, WY | AZ, CO, IN, MI, MN, VA, WY | MI | not detected at |

| Species Name | Total | Sites where Bd was sampled: # sites detected in sp. | % sites detected in sp. | # sites where not detected in sp. | % sites not detected in sp. | Countries sampled[1]: w/sites where detected in sp. | w/sites where not detected in sp. | States/Provinces sampled[2]: w/sites where detected in sp. | w/sites where not detected in sp. | State eggs collected | Bd detection (sites per species) in egg-collection state |
|---|---|---|---|---|---|---|---|---|---|---|---|
| *Pseudacris crucifer* | 33 | 6 | 18 | 27 | 82 | US | CA, US | LA, ME, TX, VA | QC, CT, GA, MI, MN, PA, SC, TN, VA | PA | 1 site in MI not detected at 5 sites in PA |
| *Pseudacris feriarum* | 1 | 0 | 0 | 1 | 100 | none | US | none | GA | NC | not previously sampled in NC |
| *Lithobates catesbeianus* | 313 | 182 | 58 | 131 | 42 | CA, US | CA, US | BC, ON, QC, AZ, CA,CT, FL, GA, IA, IL, MD, ME, MO, NC, NE, NV, OR, OK, SC, TX, VA, VT, WA, WV | ON, PE, AZ, CA, FL, GA, IA, IN, KY, LA, MA, MD, ME, MS, NC, NJ, NY, OK, OR, SC, TX, VA, WA, WI | PA | not previously sampled in PA |
| *Lithobates clamitans* | 249 | 139 | 56 | 110 | 44 | CA, US | CA, US | ON, PE, QC, AL, CT, FL, GA, IA, LA, MA, MD, ME, MN, NH, NJ, NY, PA, RI, VA, WI | ON, PE, CT, FL, GA, IN, KY, LA, MD, MI, MN, NH, NJ, NY, OK, SC, TX, WI | PA | detected in 6/10 sites in PA |
| *Lithobates pipiens* | 135 | 44 | 33 | 91 | 67 | CA, US | CA, US | AB, BC, PE, QC, CO, CT, IA, ME, MI, | PE, CO, CT, IA, MA, ME, MI, MN, MT, | PA | not previously |

| | | Sites where Bd was sampled | | | | Countries sampled[1] | | States/Provinces sampled[2] | | | |
|---|---|---|---|---|---|---|---|---|---|---|---|
| **Species Name** | Total | # sites detected in sp. | % sites detected in sp. | # sites where not detected in sp. | % sites not detected in sp. | w/sites where detected in sp. | w/sites where not detected in sp. | w/sites where detected in sp. | w/sites where not detected in sp. | State eggs collected | Bd detection (sites per species) in egg-collection state |
| | | | | | | | | MN, MT, NV, VA, VT, WA, WI, WY | NY, VA, VT, WI, WY | | sampled in PA |
| *Lithobates sphenocephalus* | 55 | 18 | 33 | 37 | 67 | US | US | AL, AR, GA, LA, NC, OK, SC, TN, VA | AL, FL, GA, KY, MO, OK, SC, TX, VA | FL | not detected at 4 sites in FL |
| *Lithobates sylvaticus* | 154 | 40 | 26 | 114 | 74 | CA, US | CA, US | PE, NT, QC, YT, AK, CT, GA, CO, ME, MI, MN, TN, VA, WY | BC, NT, PE, AK, CO, CT, GA, MA, ME, MI, MN, NJ, PA, VA, WI, WY | MI | detected in 1/2 sites in MI |
| *Rana aurora* | 42 | 12 | 29 | 30 | 71 | CA, US | CA, US | BC, CA, OR | BC, CA, OR | OR | detected in 2/24 sites in OR |
| *Rana cascadae* | 39 | 17 | 44 | 22 | 56 | US | US | CA, WA, OR | CA, WA, OR | OR | detected in 1/6 sites in OR |
| *Rana luteiventris* | 138 | 74 | 54 | 64 | 46 | US | US | ID, MT, OR, UT, WA, WY | AK, MT, OR, UT, WY | ID | deteted in 5/5 sites in ID |
| *Anaxyrus americanus* | 27 | 8 | 30 | 19 | 70 | CA, US | US | QC, CT, ME, MI, MN, VA | GA, CT, IN, MA, ME, MI, OK, RI, VA, WI | PA | not previously sampled in PA |

| Species Name | Total | Sites where Bd was sampled: # sites detected in sp. | % sites detected in sp. | # sites where not detected in sp. | % sites not detected in sp. | Countries sampled[1]: w/sites where detected in sp. | w/sites where not detected in sp. | States/Provinces sampled[2]: w/sites where detected in sp. | w/sites where not detected in sp. | State eggs collected | Bd detection (sites per species) in egg-collection state |
|---|---|---|---|---|---|---|---|---|---|---|---|
| *Anaxyrus boreas* | 444 | 195 | 44 | 249 | 56 | CA, US | CA, US | AB, BC, NT, YT, AK, CA, CO, ID, MT, OR, UT, WA, WY | BC, AK, CA, CO, ID, MT, NV, OR, UT, WA, WY | OR | detected at 21/38 sites in OR |
| *Anaxyrus fowleri* | 25 | 9 | 36 | 16 | 64 | US | US | TN, VA | CT, GA, IN, LA, MA, MS, TN, VA | NC | not previously sampled in NC |
| *Anaxyrus terrestris* | 11 | 1 | 9 | 10 | 91 | US | US | FL | FL, GA, NC, OK, SC, VA | FL | detected at 1 site sampled FL |

[1]Country codes: CA – Canada; MX – Mexico; US – United States

[2]State/Province codes: (Canada) AB – Alberta; BC – British Columbia; NT – Northwest Territories; ON – Ontario; PE – Prince Edward Island; QC – Quebec; YT – Yukon Territory; (United States) AL – Alabama; AK – Alaska; AR – Arizona; CA – California; CO – Colorado; CT – Connecticut; FL – Florida; GA – Georgia; IA – Iowa; ID – Idaho; IL – Illinois; IN – Indiana; KY – Kentucky; LA – Louisiana; MA – Massachusetts; MD – Maryland; ME – Maine; MI – Michigan; MN – Minnesota; MO – Missouri; MS – Mississippi; MT – Montana; NC – North Carolina; NE – Nebraska; NH – New Hampshire; NJ – New Jersey; NV – Nevada; NY – New York; OK – Oklahoma; OR – Oregon; PA – Pennsylvania; RI – Rhode Island; SC – South Carolina; TN – Tennessee; TX – Texas; UT – Utah; VA – Virginia; VT – Vermont; WA – Washington; WI – Wisconsin; WV – West Virginia; WY – Wyoming

[3]California samples for *Ps. regilla* may have included some animals that would now be classified as *Ps. sierra*.
