# Supplementary material for: Linking Ecology and Epidemiology to Understand Predictors of Multi-Host Responses to an Emerging Pathogen, the Amphibian Chytrid Fungus
Source: PLoS One. 2017 Jan 17;12(1):e0167882. doi: 10.1371/journal.pone.0167882 (PMC5240985; doi:10.1371/journal.pone.0167882)
Supplement: S6 Table — (PDF) [file pone.0167882.s006.pdf]

S6 Table. Amphibian species information, egg collection location, sample sizes and year of study. Year 1 = 2009; year 2 = 2010; year 3 = 2011; year 4 = 2012.

**Common Name**	**Species Name**	**Family**	**Location Collected/Collector**	**N**	**Year of study**
Pseudacris regilla	Pacific tree frog	*Hylidae*	Jefferson County, OR (Blaustein lab)	50	3
Pseudacris ornata	Ornate chorus frog	*Hylidae*	Liberty County, FL (Moriarty Lemmon lab)	30	3
Pseudacris triseriata	Western chorus frog	*Hylidae*	Livingston County, MI (Relyea lab)	50	1
Pseudacris crucifer	Spring peeper	*Hylidae*	Crawford County, PA (Relyea lab)	50	2
Pseudacris feriarum	Upland chorus frog	*Hylidae*	Knox County, NC (Price lab)	50	1
Hyla squirella	Squirrel tree frog	*Hylidae*	Orange County, FL (Moriarty Lemmon lab)	35	3
Hyla wrightorum	Arizona tree frog	*Hylidae*	Coconino County, AZ (Collins lab)	49	3
Hyla versicolor	Gray tree frog	*Hylidae*	Crawford County, PA (Relyea lab)	50	1
Lithobates clamitans	Green frog	*Ranidae*	Crawford County, PA (Relyea lab)	20	3
Rana cascadae	Cascades frog	*Ranidae*	Deschutes County, OR (Blaustein lab)	31	3
Rana luteiventris	Columbia spotted frog	*Ranidae*	Latah County, ID (Caren Goldberg)	49	3
Lithobates pipiens	Northern leopard frog	*Ranidae*	Crawford County, PA (Relyea lab)	50	1
Lithobates sphenocephalus	Southern leopard frog	*Ranidae*	Wakulla County, FL (Susan Walls)	50	3
Lithobates sylvaticus	Wood frog	*Ranidae*	Livingston County, MI (Relyea lab)	44	1
Rana aurora	Red-legged frog	*Ranidae*	Lane County, OR (Blaustein lab)	43	3
Lithobates catesbeianus	American bullfrog	*Ranidae*	Crawford County, PA (Relyea lab)	33	4
Anaxyrus americanus	American toad	*Bufonidae*	Crawford County, PA (Relyea lab)	50	2
Anaxyrus terrestris	Southern toad	*Bufonidae*	Orange County, FL (Fauth lab)	50	1
Anaxyrus fowleri	Fowler's toad	*Bufonidae*	Mecklenburg County, NC (Price lab)	47	2
Anaxyrus boreas	Western toad	*Bufonidae*	Deschutes County, OR (Blaustein lab)	41	3
