# Supplementary material for: Linking Ecology and Epidemiology to Understand Predictors of Multi-Host Responses to an Emerging Pathogen, the Amphibian Chytrid Fungus
Source: PLoS One. 2017 Jan 17;12(1):e0167882. doi: 10.1371/journal.pone.0167882 (PMC5240985; doi:10.1371/journal.pone.0167882)
Supplement: S7 Table — (PDF) [file pone.0167882.s007.pdf]

S7 Table. Ecological and life-history summaries for species in experimental study. Information about habitat use and breadth was collected by surveying scientists that have published papers on the focal species in this study. Geographic range area estimates were obtained from [82]. All other ecological and life history traits were collected at the species level by surveying 2 amphibian references [76-77] and 4 global databases [78-81]. We collected all information available from sources where species accounts were present for the traits of interest and combined them to obtain a median estimate of each trait for each species.

**Species Name**	**Habitat Use (scale 1-5)***	**Habitat Breadth (scale 1-3)****	**Median adult body size (snout-vent-length in mm)**	**Median larval period (weeks)**	**Median age at sexual maturity (months)**	**Median lifespan (years)**	**Median number of eggs laid per year**	**Geographic range size (km2)**
Anaxyrus americanus	2.72	2.58	80	1.75	48	10	10850	5600000
Anaxyrus boreas	3.2	2.6	100	2	48	8.5	9750	347500
Anaxyrus fowleri	2.8	2.79	68.5	1.5	24	4.5	9000	2035000
Anaxyrus terrestris	2.92	2.89	76	1.5	36	9	3250	547500
Hyla squirella	2.33	1.95	33	1.5	18	8.5	1000.5	730000
Hyla versicolor	2.86	2.25	46	1.5	24	8	1800	2265000
Hyla wrightorum	2	1.85	37.5	2	18	8.25	75	145000

Lithobates catesbeianus	4.22	1.54	116	19.5	36	8.5	24,743.5	4457500
Lithobates clamitans	3.83	2.03	80	19	36	5.5	4000	3672500
Lithobates pipiens	3.53	1.95	80.5	4	24	6.5	3974	5945000
Lithobates sphenocephalus	3.38	2.2	75	2.75	20	4	3277	1965000
Lithobates sylvaticus	2.78	2.11	60	3	24	4	1650	40000
Pseudacris crucifer	2.96	2.32	29	2.25	24	3	890.5	3982500
Pseudacris feriarum	2.72	2.33	29.5	3.25	18	2	831.5	1430000
Pseudacris ornata	2.32	1.58	32.5	3.5	12	2.25	58	367500
Pseudacris regilla	2.92	2.47	35	2.5	18	3	575	1575000
Pseudacris triseriata	2.59	2.29	24.5	2.5	18	5	1000	3485000
Rana aurora	3.29	2.26	88	5	24	11.5	750	292500
Rana cascadae	3.46	2	65	2	48	6	550	115000
Rana luteiventris	3.4	2.25	70	4.5	42	8	1275	1315000

* Habitat use scale spanned from 1 (highly ephemeral) to 5 (permanent): (1) Dries within weeks with no or few small invertebrate predators, (2) dries every year with few small invertebrate predators, (3) dries every few years and occasionally has large invertebrate predators, (4) never dries and has large invertebrate predators, (5) never dries and has fish and large invertebrate predators.

** Habitat breadth spanned from 1 (only 1 type of habitat occupied) to 3 (the species exists in all habitats, or exhibits broader habitat usage across a range of habitat types).
