# Supplementary material for: Linking Ecology and Epidemiology to Understand Predictors of Multi-Host Responses to an Emerging Pathogen, the Amphibian Chytrid Fungus
Source: PLoS One. 2017 Jan 17;12(1):e0167882. doi: 10.1371/journal.pone.0167882 (PMC5240985; doi:10.1371/journal.pone.0167882)

S1 Figure. Non-linear relationships from GAM analysis. There were non-linear relationships between (**A**) geographic range size and average infection load, (**B**) lifespan and infection load, (**C**) average mass at metamorphosis and infection load, (**D**) eggs laid per year and hazard ratio and (**E**) geographic range size and hazard ratio.

A.

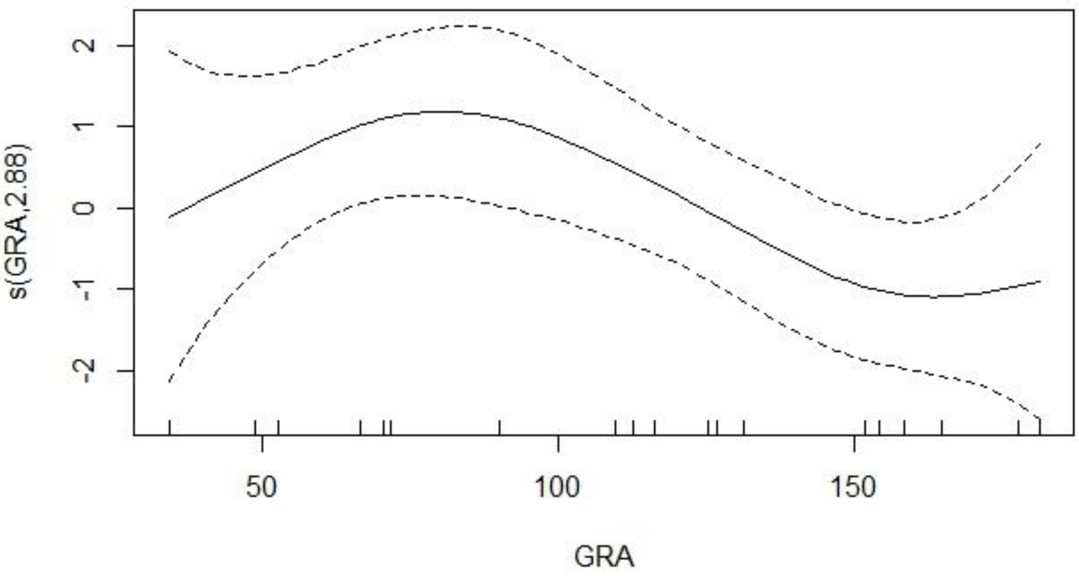

B.

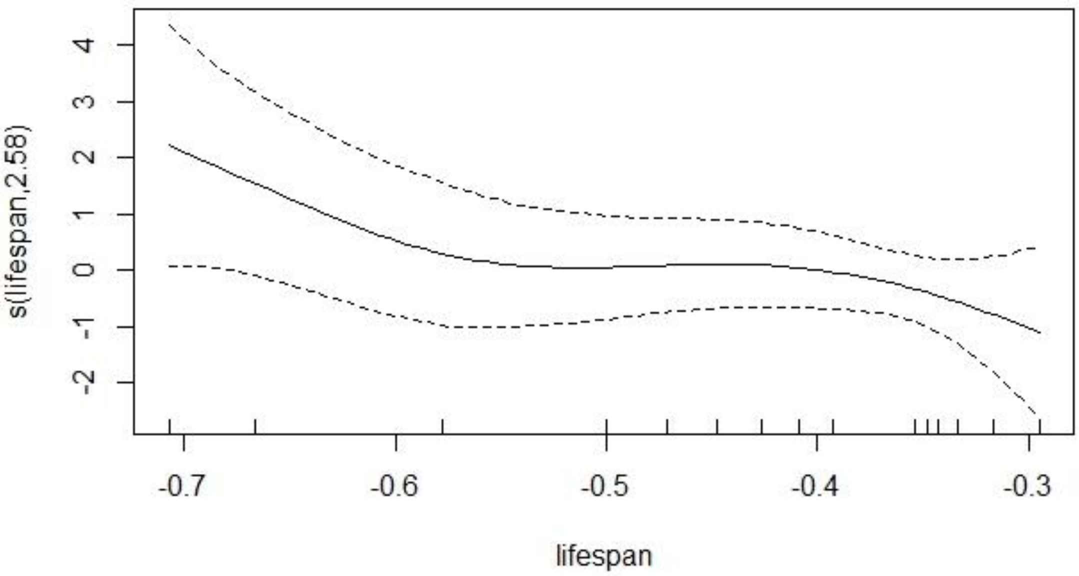

C.

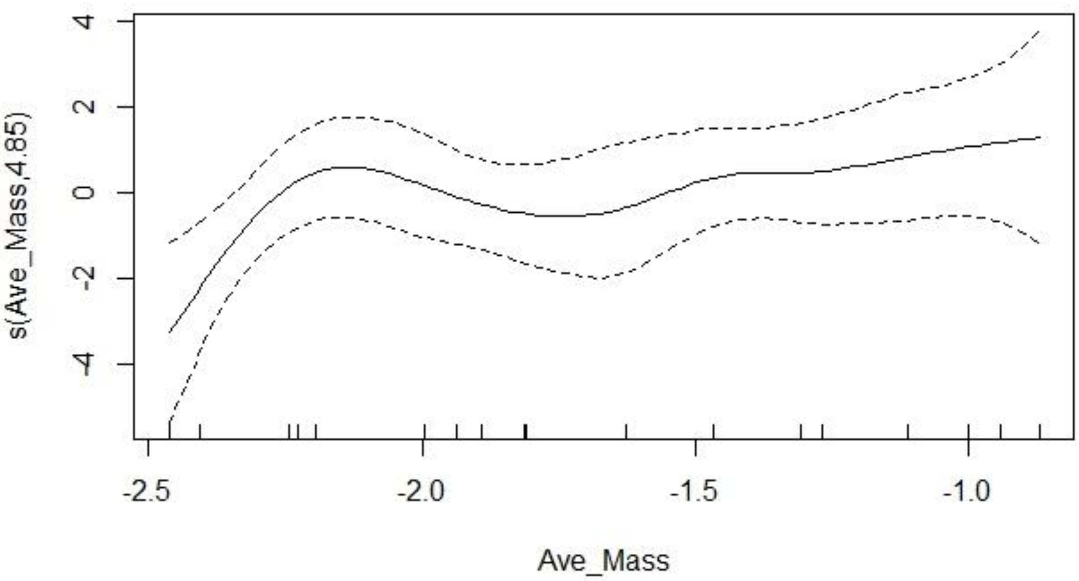

D.

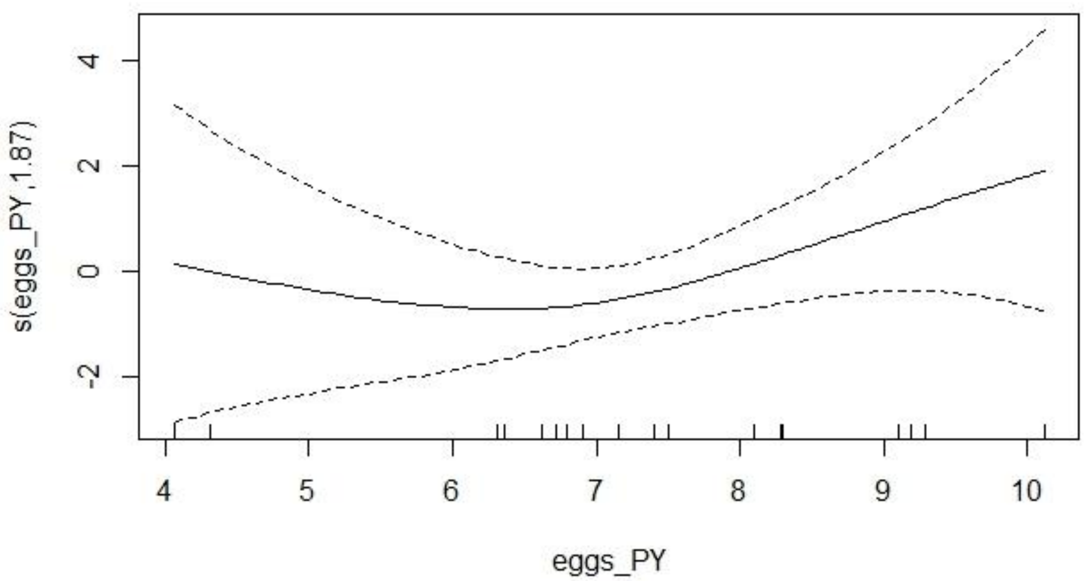

E.

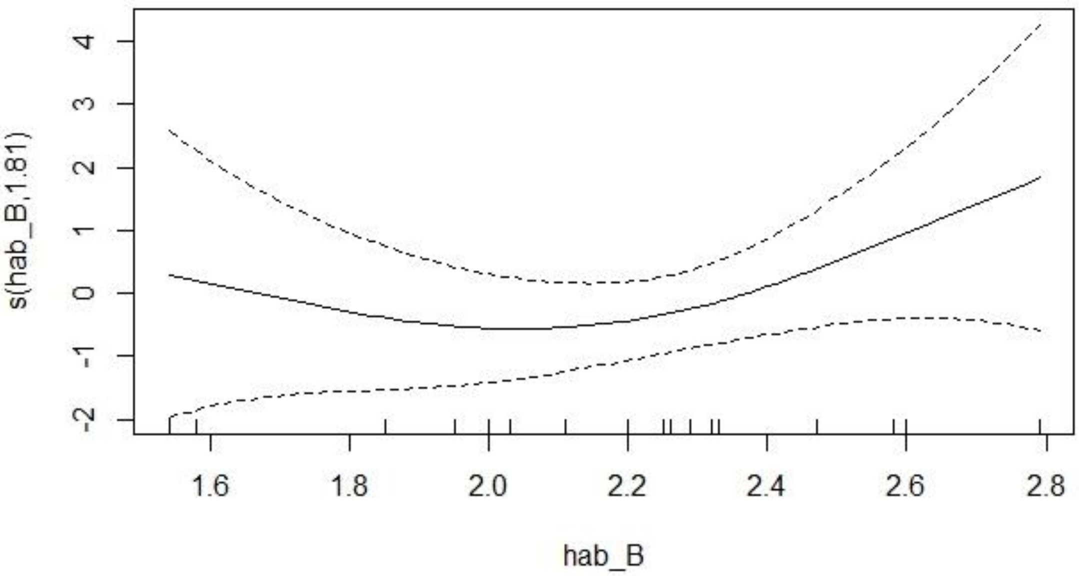

Supplement: S1 Fig — (PDF) [file pone.0167882.s008.pdf]
